# Medial soft tissue release is also related to the anterior stability of cruciate-retaining total knee arthroplasty: a cadaveric study

**DOI:** 10.1186/s43019-024-00233-6

**Published:** 2024-10-08

**Authors:** Sayako Sakai, Shinichiro Nakamura, Takahiro Maeda, Shinichi Kuriyama, Kohei Nishitani, Yugo Morita, Yugo Morita, Yusuke Yamawaki, Yuki Shinya, Shuichi Matsuda

**Affiliations:** Department of Orthopaedic Surgery, Graduate School of Medicine, Kyoto University, 54 Shogoin-Kawaharacho, Sakyo-Ku, Kyoto, 606-8507 Japan

**Keywords:** Knee, Total knee arthroplasty, Ligament balancing, Medial release, Mediolateral gap, Anterior posterior stability

## Abstract

**Background:**

Medial soft tissue release is occasionally performed to achieve mediolateral ligament balance in total knee arthroplasty (TKA), whose sequential effect on mediolateral and anteroposterior stability remains unclear. This study aimed to quantitatively evaluate the difference in mediolateral and anteroposterior stability according to a sequential medial soft tissue release in TKA.

**Methods:**

Cruciate-retaining TKA was performed in six cadaveric knees. Medial and lateral joint gaps, varus-valgus angle, and tibial anterior and posterior translations relative to the femur with pulling and pushing forces, respectively, were measured. All measurements were performed at full extension and 45° and 90° flexion after release of the deep medial collateral ligament (MCL) (stage 1), the posteromedial capsule (stage 2), and the superficial MCL (stage 3). Mediolateral and anteroposterior stability were compared between stages, and correlations between mediolateral and anteroposterior stability were analyzed.

**Results:**

Medial joint gap significantly increased from stages 1 to 3 by 3.2 mm, 6.8 mm, and 7.2 mm at extension, 45° flexion, and 90° flexion, respectively, and from stages 2 to 3 by 3.5 mm at extension. Varus-valgus angle was varus at stage 2, which turned to valgus at stage 3 (−2.7° to 0.8°, −2.2° to 4.3°, and −5.5° to 2.5° at extension, 45° flexion, and 90° flexion, respectively). Anterior translation at 90° flexion significantly increased from stages 1 and 2 to stage 3 by 11.5 mm and 8.2 mm, respectively, which was significantly correlated with medial gap (*r* = 0.681) and varus-valgus angle (*r* = 0.495).

**Conclusions:**

Medial soft tissue release also increased tibial anterior translation as well as medial joint gap, and medial joint gap and tibial anterior translation were significantly correlated. Surgeons should be careful not to create too large medial joint gap and tibial anterior translation in flexion by excessive medial release up to the superficial MCL for achieving an equal mediolateral joint gap in extension.

## Background

Medial soft tissue release is occasionally performed during total knee arthroplasty (TKA) for severe varus knees to equalize the medial and lateral joint gaps. However, a loose medial joint gap due to over-release worsened clinical outcomes [1–3] and increased femoral anteroposterior translation, also resulting in inferior patient-reported outcome measures [4–6]. Management to avoid a loose medial joint gap is important for successful TKA.

Recently, a medial stabilizing technique that minimizes medial soft tissue release to the deep medial collateral ligament (dMCL) has been proposed to achieve medial stability, and a larger lateral than medial joint gap is tolerated [7–11]. In a medial stabilizing technique, proper tension, or near-normal stability of the medial collateral ligament (MCL) and posterior cruciate ligament (PCL), is achieved by cutting adequate amounts of bone from the femur and tibia, which requires no medial release. In addition, extensive medial release to achieve an equal mediolateral extension gap is not recommended, because this procedure would cause too large medial flexion gap [12]. However, it is impossible to intraoperatively assess how far further release contributes to the medial and lateral joint gaps and anteroposterior translations by releasing in sequence to achieve optimal stability.

Medial release increased medial joint gap in flexion and extension [5, 7], and medial joint gap is related to anteroposterior stability [8–10]. However, sequential evaluation of an effect of medial soft tissue release on mediolateral and anteroposterior stability throughout range of motion following the actual surgical procedure in TKA has not been investigated. The present study aimed to quantitatively evaluate the extent to which and at what flexion angle medial and lateral joint gaps and tibial anteroposterior translation relative to the femur increase by sequential medial soft tissue release in cruciate-retaining (CR) TKA using cadaveric knees, and to investigate an effect of mediolateral stability on anteroposterior stability. It was hypothesized that both the medial gap and tibial anterior translation relative to the femur would increase after the superficial MCL (sMCL) release at 90° flexion, and that larger medial gap would increase tibial anterior translation relative to the femur.

## Material and methods

### Specimens

Three pairs of cadaveric knees from one female and two male Thiel-embalmed full-body specimens were examined. All of the cadavers were donated to our institute voluntarily, and the present study was approved by our institutional review board. The ages at death were 62, 65, and 81 years old. None of the knees had macroscopic signs of osteoarthritis (OA), previous trauma, or knee surgery. Preoperative whole-limb alignment was measured using a post mortem computed tomography: the weight-bearing line ratio (WBLR), a percentage of the intersection of the weight-bearing line and the tibial plateau (medial and lateral edge were defined as 0% and 100%, respectively); hip-knee-ankle angle (HKAA), an angle between the femoral and tibial mechanical axes; mechanical lateral distal femoral angle (mLDFA), a lateral angle between the femoral mechanical axis and a line tangent to the distal femur; and medial proximal tibial angle (MPTA), a medial angle between the tibial mechanical axis and a line tangent to the tibial plateau surface, presented in Table 1.

**Table 1:** Lower limb alignment of each specimen

			WBLR (%)	HKAA (°)	mLDFA (°)	MPTA (°)
1	Female	Right	30.5	−4.5	87.4	87.0
2	Female	Left	42.0	−2.1	85.6	86.0
3	Male	Right	32.0	−3.9	88.6	86.0
4	Male	Left	41.1	−2.2	82.8	84.5
5	Male	Right	26.6	−2.6	87.6	84.1
6	Male	Left	32.4	−2.1	87.7	86.3
Mean			34.1	−2.9	86.6	85.7
SD			6.1	1.0	2.1	1.1

WBLR, weight-bearing line ratio; HKAA, hip-knee-ankle angle; mLDFA, mechanical lateral distal femoral angle; MPTA, medial proximal tibial angle; SD, standard deviation

### Surgical technique

CR-TKA was performed with a standard midline skin incision and medial parapatellar arthrotomy using the Initia Total Knee System CR Type (Kyocera, Kyoto, Japan). No additional medial soft tissue release was performed except the dMCL, which was detached subperiosteally 10 mm distal to the medial joint line. The distal femur was cut perpendicular to the femoral mechanical axis in the coronal plane and parallel to the distal femoral anatomical axis in the sagittal plane. The axial rotation of the femoral component was aligned with the surgical epicondylar axis. The femoral component size was determined on the basis of a posterior reference. The proximal tibia was cut perpendicular to the tibial mechanical axis in the coronal plane approximately 10 mm from the most proximal surface of the lateral tibial plateau, retaining the PCL attachment as a bone island. A posterior tibial slope of 7° relative to the tibial shaft was set in the sagittal plane. Tibial rotational alignment was aligned with the line connecting the tibial insertion of posterior cruciate ligament and medial margin of the patellar tendon [13]. In this analysis, the same insert thickness (9 mm) was used in all TKAs. The patella was not resurfaced.

### Measurements

A series of medial soft tissue releases and measurements were performed in sequence. The medial soft tissue release was performed in the dMCL, posteromedial capsule (PMC), and sMCL. The medial and lateral joint gaps, varus-valgus angle, and tibial anterior and posterior translations relative to the femur were measured using actual surgical measurement devices at 0°, 45°, and 90° flexion. The measurements were conducted in three stages; firstly after the procedure with the release of dMCL mentioned above (stage 1), secondly after the additional release of PMC (stage 2), and finally after the additional release of sMCL (stage 3). The PMC was detached subperiosteally from the tibial attachment using a scalpel, and the sMCL was detached subperiosteally from its proximal tibial attachment using a periosteal elevator, and sharply resected just above the distal tibial insertion using a scalpel.

**Fig. 1:**
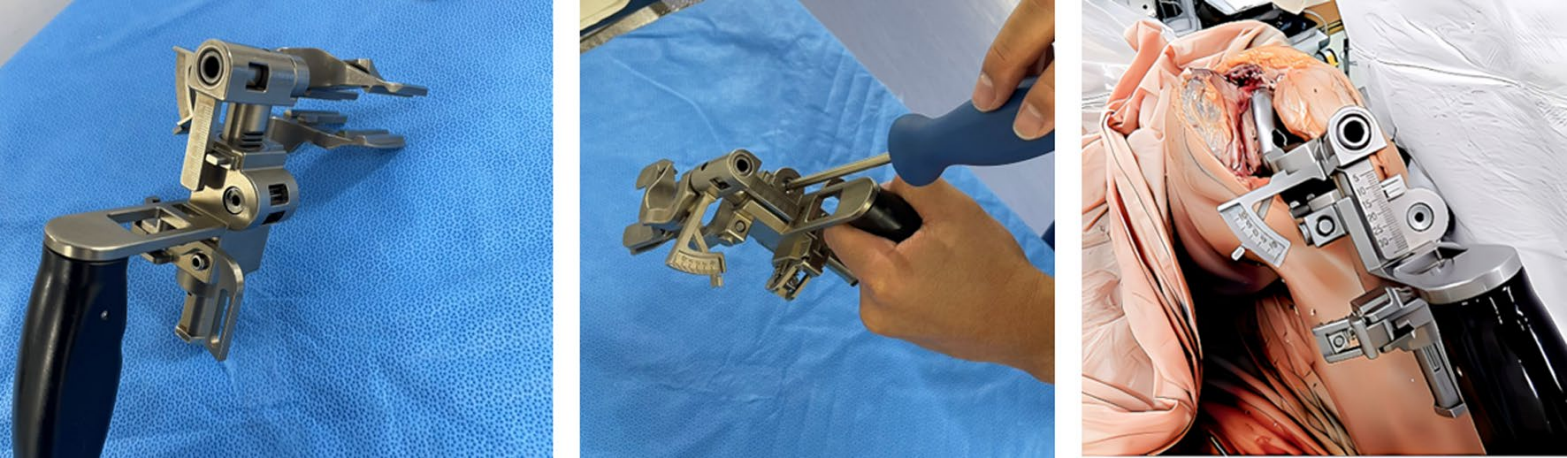
The joint center gap and varus-valgus angle were measured using a tensor device (left). A distraction force of 40 lb (178 N) was applied with a torque screwdriver (center).The tensor device shows the joint center gap of 15 mm with 1° varus (right)

The center gap and varus-valgus angle were measured manually without any measurable device such as a navigation system at 0°, 45°, and 90° flexion. A distraction force of 40 lb (178 N) was applied using an offset-type tensor device with the trial femoral component (Fig. 1) [9–11, 14]. Medial and lateral gaps were calculated on the basis of the center gap, varus-valgus angle, and transverse diameter of the tibial plate using trigonometric function as per previous studies [15, 16]. Measurements were performed three times to confirm the measured gap remaining constant, and the third measured value was adopted to reduce errors due to creep elongation of surrounding soft tissues [17, 18]. For the varus-valgus angle, valgus was denoted as positive.

**Fig. 2:**
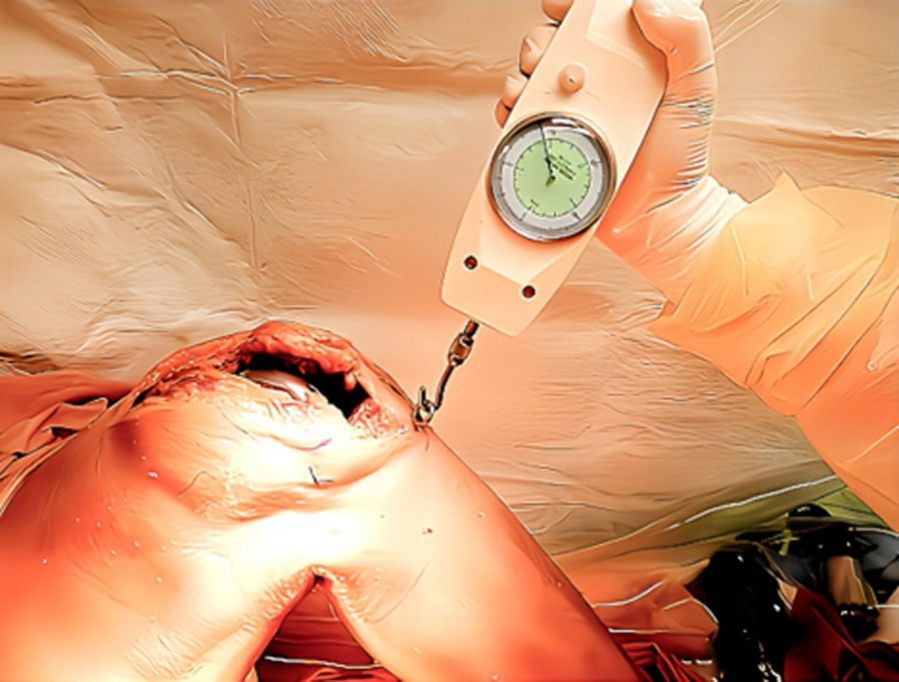
Tibial anterior and posterior translations relative to the femur were measured applying pulling and pushing forces through an eyelet screw fixed to the tibial tuberosity using a handheld analog force gauge. The figure shows applying a pulling force for the measurement of tibial anterior translation

Tibial anterior and posterior translations relative to the femur were measured using a paddle-type device after fixing the trial tibial component and polyethylene insert. The device was fixed to the anterior surface of the tibial tray, and the paddle part of the device was set on the medial part of the femoral component, which could measure the position of the anterior margin of the tibial tray from the medial condyle of the femoral component with a built-in ruler. The zero position was defined as no force applied to the tibia. The tibial positions were measured while applying pulling and pushing forces of 70 N through an eyelet screw fixed to the tibial tuberosity using a handheld analog force gauge (Fig. 2) [10]. The measurements with pulling and pushing forces were conducted five times each, and the averages of the three intermediate values were used for data. The tibial translations with pulling and pushing forces relative to the zero position were defined as “tibial anterior translation” and “tibial posterior translation,” respectively. Each additional release was performed by removing the trial implants after completion of measurements at the previous stage. During each measurement, the thigh was held by an assistant and aligned in the sagittal plane with the hip and ankle joints in neutral rotation to eliminate external load on the knee. The intrarater reliabilities were assessed using intraclass correlation coefficients for both anterior and posterior translations, which were > 0.92 and > 0.81, respectively.

### Statistical analysis

All statistical analyses were performed using the R software (version 4.2.1; R Foundation for Statistical Computation, Vienna, Austria). Normality was examined using the Shapiro–Wilk test for each group used for comparison. Comparisons of mediolateral and anteroposterior laxity among the stages were conducted using one-way repeated measures analysis of variance (RM ANOVA) for normal distributions. Greenhouse–Geisser correction was used for violations of the sphericity according to Mauchly’s sphericity test. Friedman’s test was used for non-normal distributions. Post hoc multiple comparisons were performed using Shaffer’s modified sequentially rejective multiple-test procedure. To investigate the correlation between mediolateral and anterolateral stabilities, Pearson’s correlation test and Spearman’s rank correlation test were performed for normal and non-normal distributions, respectively. Statistical significance level was set at *P*-value < 0.05. Post hoc power analysis was performed in relation to the tibial anterior translation at 90° of knee flexion on the basis of a significant level of *α* < 0.05 using G*power 3.1.9.7 (Heinrich-Heine-Universität Düsseldorf, Düsseldorf, Germany). An effect size η^2^ of 0.54 and power (1-β) of 0.99 were obtained.

## Results

### Medial and lateral gaps and varus-valgus angle

**Fig. 3:**
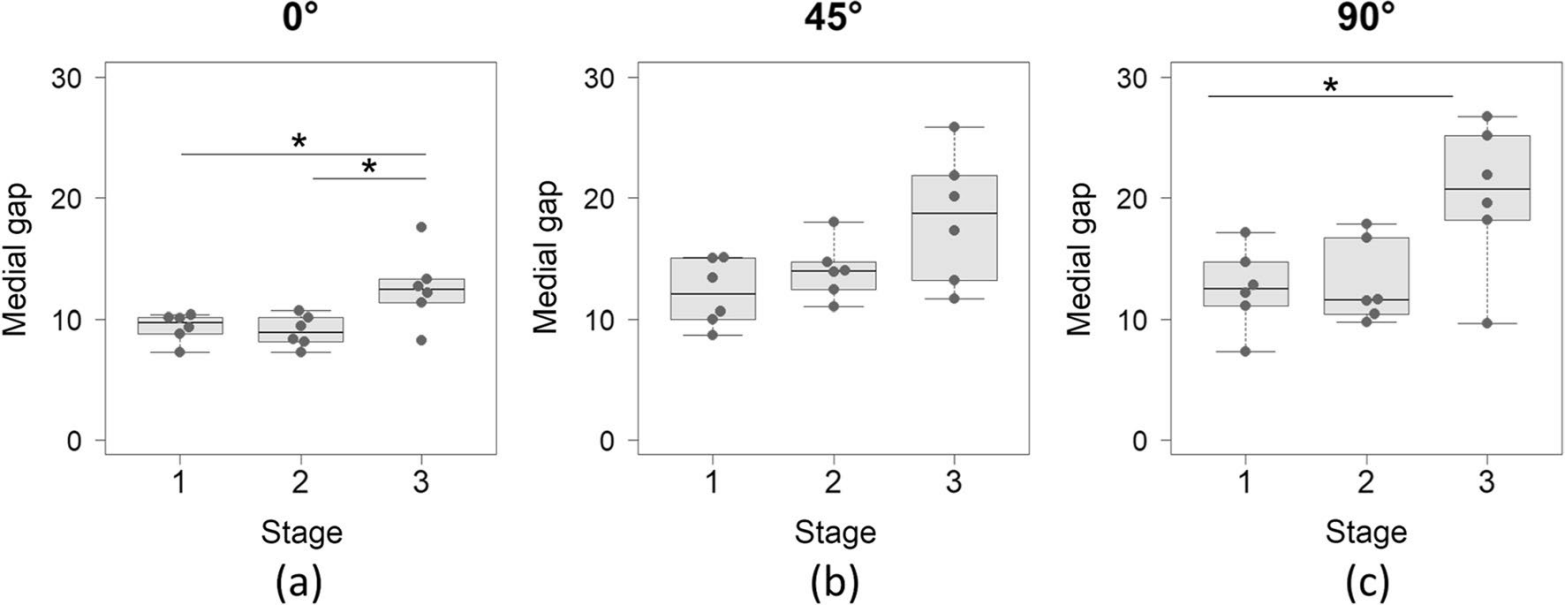
(**a**) Medial gap at 0° flexion was significantly larger at stage 3 than at stage 1 and stage 2. (**b**) There was no significant difference of medial gap at 45° flexion between the stages by a post hoc multiple comparison. (**c**) Medial gap at 90° flexion was significantly larger at stage 3 than at stage 1

**Fig. 4:**
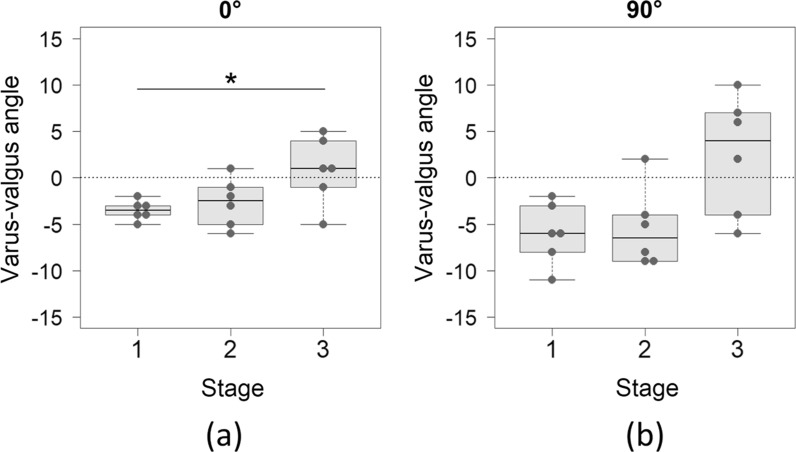
(**a**) Varus-valgus angle at 0° flexion was significantly larger at stage 3 than at stage 1. (**b**) There was no significant difference of varus-valgus angle at 90° flexion between the stages by a post hoc multiple comparison. At both flexion angles, varus angles at stage 1 and stage 2 turned valgus at stage 3

Medial gap significantly differed among the stages by one-way RM ANOVA in all knee flexion angles (Table 2). Post hoc multiple comparison showed that medial gap significantly increased at stage 3 from stage 2 at 0° flexion (Fig. 3a), and from stage 1 at 90° flexion (Fig. 3c), but there was no significant difference at 45° flexion (Table 2 and Fig. 3b). Lateral gap was not significantly different among the stages in all knee flexion angles (Table 2). Varus-valgus angles were varus at stage 1 and stage 2, which turned to valgus at stage 3 (Table 2 and Fig. 4). At 0° flexion, there was a significant difference of varus-valgus angle between stage 1 and stage 3 by post hoc multiple comparison (Fig. 4a). At 90° flexion, there were no significant differences between the stages, although one-way RM ANOVA showed significance (Table 2 and Fig. 4b).

**Table 2:** Medial and lateral gaps, varus-valgus angle, and tibial anterior and posterior translations per knee flexion angle and stage

		Stage 1	Stage 2	Stage 3	*P*-value	Post hoc multiple comparison		
						1 versus 2	2 versus 3	1 versus 3
Medial gap (mm)	**0°**	**9.3 ± 1.2**	**9.0 ± 1.3**	**12.5 ± 3.0**	0.002^*^	0.582	**0.033**	**0.033**
	**45°**	**12.1 ± 2.7**	**14.0 ± 2.4**	**18.3 ± 5.4**	0.026^*^	0.252	0.118	0.082
	**90°**	**12.5 ± 3.3**	**13.0 ± 3.4**	**20.2 ± 6.1**	**0.048**^**†**^	0.827	0.083	**0.018**
Lateral gap (mm)	**0°**	11.5 ± 0.8	10.7 ± 2.0	12.1 ± 1.7	0.119^*^			
	**45°**	12.9 ± 1.5	15.3 ± 2.4	16.0 ± 3.4	0.067^*^			
	**90°**	16.3 ± 5.5	16.4 ± 4.1	18.8 ± 5.3	0.109^*^			
Varus-valgus angle (°)	**0°**	**−3.5 ± 1.0**	**−2.7 ± 2.6**	**0.8 ± 3.6**	0.025^*^	0.418	0.121	**0.046**
	**45°**	**−2.9 ± 1.9**	**−2.2 ± 3.8**	**4.2 ± 7.2**	0.042^*^	0.626	0.171	0.171
	**90°**	**−6.0 ± 3.3**	**−5.5 ± 4.2**	**2.5 ± 6.4**	0.049^*^	0.610	0.074	0.071
Anterior translation (mm)	**0°**	3.1 ± 1.2	4.7 ± 2.4	7.4 ± 6.9	0.227^*^			
	**45°**	10.6 ± 4.8	10.7 ± 5.3	19.0 ± 12.6	0.093^*^			
	**90°**	**9.8 ± 4.4**	**13.1 ± 4.1**	**21.3 ± 6.1**	0.005^*^	0.342	**0.043**	**0.004**
Posterior translation (mm)	**0°**	3.4 ± 4.4	−0.8 ± 1.2	−0.3 ± 0.5	0.129^†^			
	**45°**	2.0 ± 2.0	−3.9 ± 2.9	−3.8 ± 4.8	0.311^†^			
	**90°**	3.0 ± 0.3	−4.4 ± 3.8	−4.3 ± 4.4	0.821^*^			

Data are presented as mean ± standard deviation. Bold texts indicate statistically significant difference

^*^One-way repeated measures analysis of variance

^†^Friedman’s test

### Anterior and posterior translation

**Fig. 5:**
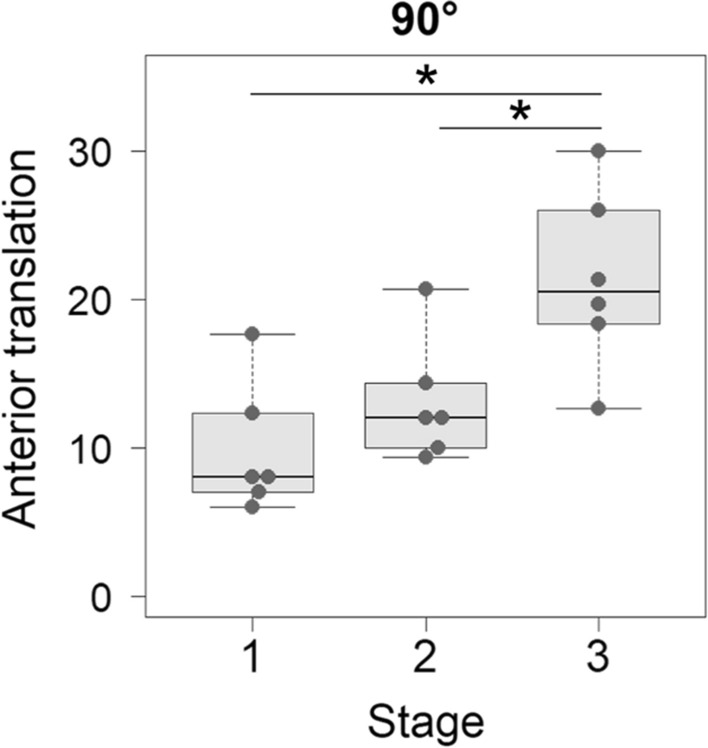
Comparisons of tibial anterior translation between the stages showing a significance by the repeated measures analysis of variance among the three stages. Tibial anterior translation at 90° flexion was significantly larger at stage 3 than at stage 1 and stage 2

Tibial anterior translation significantly differed among the stages by one-way RM ANOVA only at 90° flexion (Table 2). Post hoc multiple comparison showed that tibial anterior translation significantly increased at stage 3 from stage 1 and from stage 2 at 90° flexion (Fig. 5). Stage 3 release equalized the medial and lateral gaps at 0° flexion, with causing markedly greater tibial anterior translation at 90° flexion. Tibial posterior translation was not significantly different among the stages in all knee flexion angles (Table 2).

### Correlation between the mediolateral gap and anteroposterior translation

**Fig. 6:**
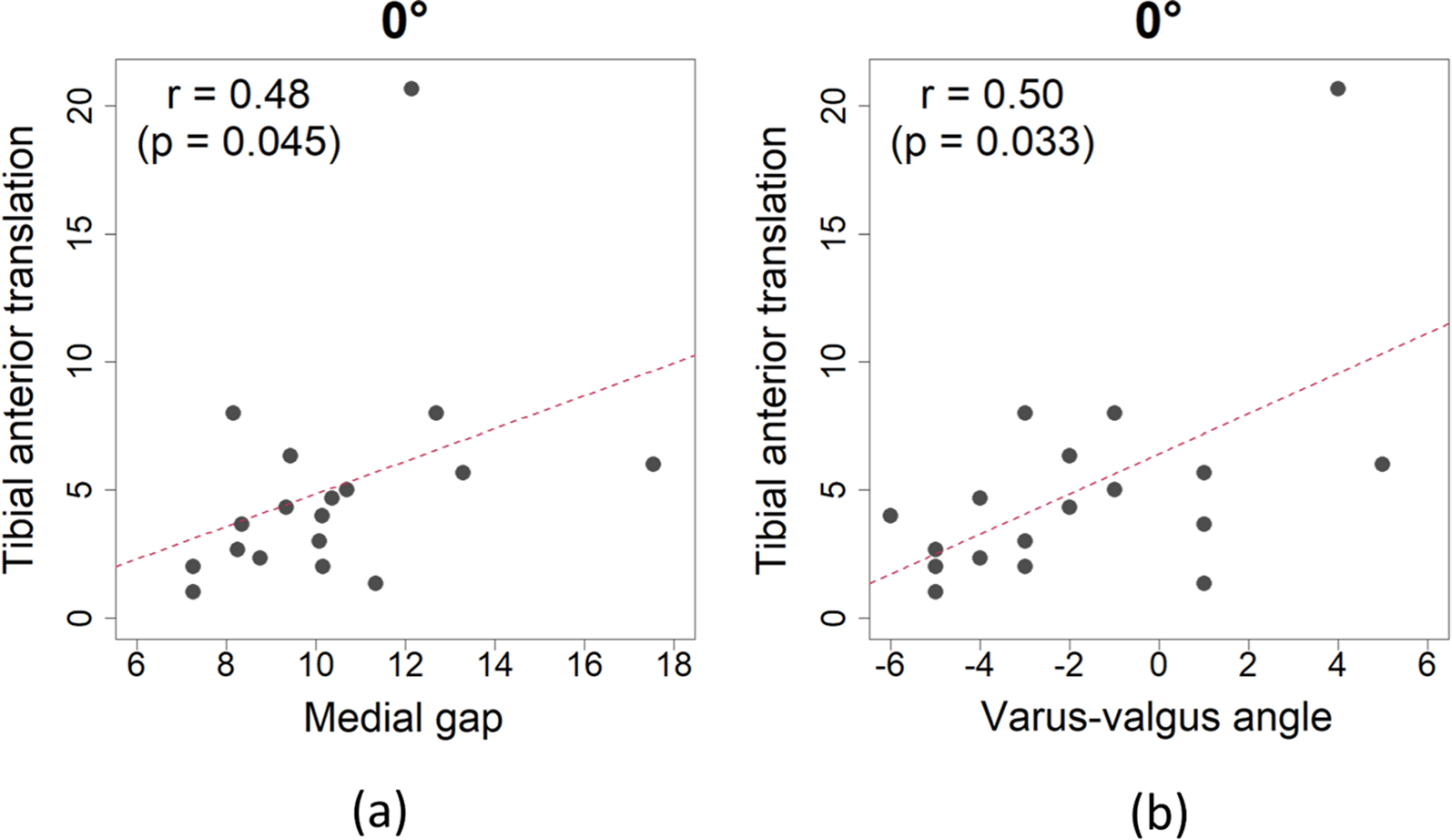
Tibial anterior translation had significant positive correlations with medial gap (**a**) and varus-valgus angle (**b**) at 0° flexion

**Fig. 7:**
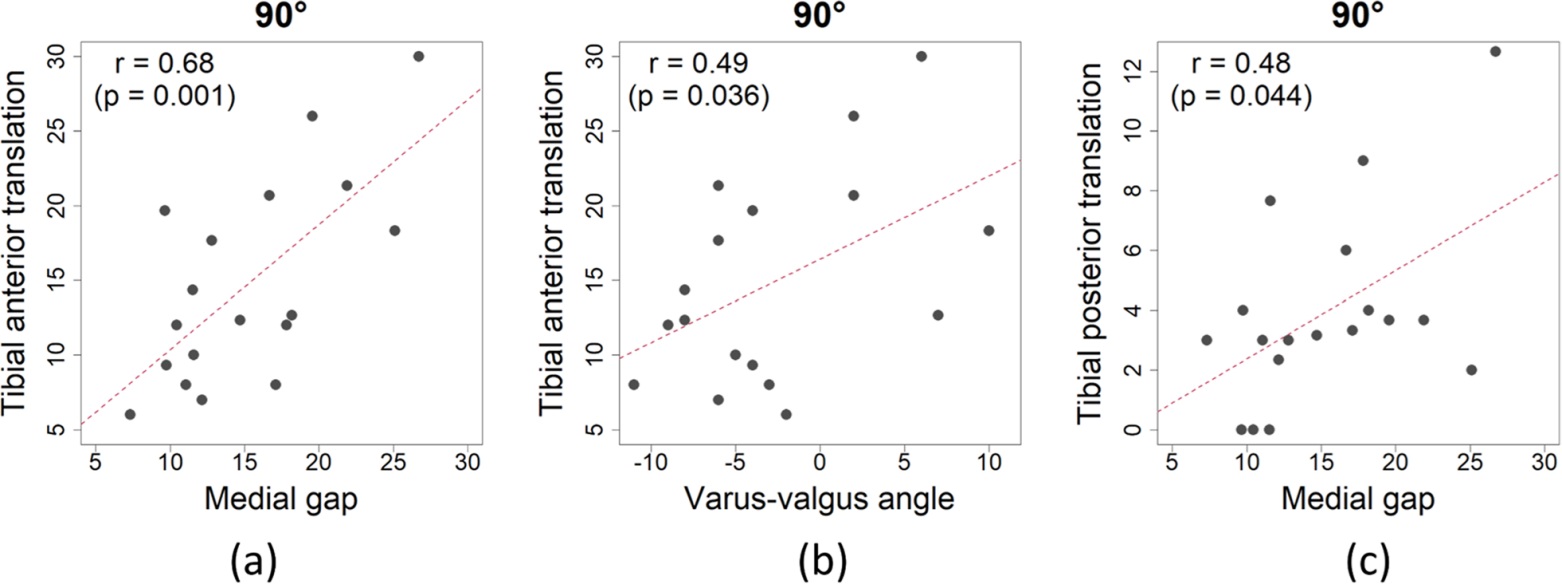
Tibial anterior translation had significant positive correlations with medial gap (**a**) and varus-valgus angle (**b**), and tibial posterior translation had a significant positive correlation with medial gap (**c**) at 90° flexion

As presented in Table 3, at 0° and 90° flexion, tibial anterior translation had significant positive correlations with medial gap (Figs. 6a and 7a) and varus-valgus angle (Figs. 6b and 7b). At 90° flexion, tibial posterior translation had a significant positive correlation with medial gap (Table 3 and Fig. 7c). There were no significant correlations between mediolateral and anterolateral stabilities at 45° flexion (Table 3). Tibial anterior translation at 0° and 90° flexion increased as medial gap increased and as the knee joint varus-valgus angle became valgus. There were no factors correlated with lateral gap.

**Table 3:** Correlations between mediolateral and anteroposterior stabilities

	0°				45°				90°			
	Anterior translation		Posterior translation		Anterior translation		Posterior translation		Anterior translation		Posterior translation	
	Coefficient	*P*-value^*^	Coefficient	*P*-value^*^	Coefficient	*P*-value	Coefficient	*P*-value^†^	Coefficient	*P*-value^*^	Coefficient	*P*-value^†^
Medial gap	**0.48**	**0.045**	−0.04	0.861	−0.18	0.469^*^	0.42	0.082	**0.68**	**0.001**	**0.48**	**0.044**
Lateral gap	0.03	0.894	0.27	0.283	−0.23	0.362^†^	0.42	0.085	0.40	0.102	0.21	0.408
Varus-valgus angle	**0.50**	**0.033**	−0.33	0.179	−0.03	0.915^*^	0.26	0.302	**0.49**	**0.036**	0.21	0.394

Bold texts indicate statistically significant correlations

^*^Pearson’s correlation test

^†^Spearman’s rank correlation test

## Discussion

The present study evaluated medial and lateral gaps, varus-valgus angle, and tibial anterior and posterior translations relative to the femur at 0°, 45°, and 90° flexion by the sequential release of the dMCL, PMC, and sMCL in CR-TKA to simulate actual surgery using cadaveric knees. The sMCL release markedly increased medial gap and tibial anterior translation at 90° flexion, although the nearly equal medial and lateral gaps and almost parallel varus-valgus angle, so-called a rectangular gap, were obtained at 0° flexion. Tibial anterior and posterior translations had significant positive correlations with medial gap. Our hypotheses that both medial gap and tibial anterior translation would increase after the sMCL release at 90° flexion and that larger medial gap would increase tibial anterior translation relative to the femur were reinforced by the results. Surgeons should be careful not to perform excessive medial release up to the sMCL to obtain a rectangular extension gap to avoid medial and anteroposterior instabilities at flexion.

It is well known that release of the sMCL, the primary restraint to valgus rotation of the knee, during TKA increases medial looseness [5, 19, 20], which are more pronounced at 90° flexion than at extension [5, 21, 22]. In the present study, medial gap significantly increased from stage 1 to stage 3 at 0° flexion (3.2 mm), but the increment was considerably larger at 90° flexion (7.2 mm). Robinson et al. cut the dMCL, PMC, and sMCL on six cadaveric native knees in a similar order to the present study, and the sMCL cut after cutting the dMCL and PMC significantly increased valgus angle by up to 8° at 0° to 90° flexion, whereas cutting the dMCL alone or additional PMC cut had no significant effect on valgus laxity at any flexion angle [23]. Iizawa et al. showed that release of the dMCL and PMC did not change medial gap in CR-TKA using cadaveric knees [7]. These previous reports support our results, which also indicated no significant increase of medial gap and varus-valgus angle from stage 1 to stage 2.

Excessive medial soft tissue release increases anterior as well as medial instabilities [9, 10, 24]. In anterior cruciate ligament (ACL)-deficient cadaveric knees, the sMCL cut after cutting the dMCL and PMC dramatically increased tibial anteromedial translation and anteromedial rotation angle as well as valgus rotation angle at 0° to 90° flexion [25]. This situation is considered similar to that in CR-TKA. While the ACL is the absolute primary restraint to tibial anterior translation, the sMCL plays a key role in preventing anteromedial instability of the knee in the absence of the ACL function. The present study found that tibial anterior translation significantly increased at stage 3 compared with stage 1 and stage 2 at 90° flexion. In addition, at 0° and 90° flexion, tibial anterior translation was significantly correlated with medial gap and varus-valgus angle, which was substantiated by previous studies which intraoperatively examined the medial gap and tibial anteroposterior translation [10, 26]. However, the effect of intraoperative release of the sMCL was not evaluated due to the need for an appropriate ligament balance in vivo. This study could release the medial soft tissue in sequence up to sMCL using cadaveric knees to simulate surgery and could evaluate the effect of excessive medial release. It is noteworthy that our results were obtained according to an actual surgical procedure.

The present study showed that at stage 3, medial gap was nearly equal to lateral gap (12.5 mm and 12.2 mm, respectively) and varus-valgus angle was almost parallel (0.8°) at 0° flexion, indicating that a rectangular gap could be finally achieved after complete release of the sMCL at extension, which caused markedly increased medial gap and tibial anterior translation at 90° flexion (11.5 mm increase from stage 1 to stage 3). The knee joint presents maximum stiffness at extension due to stretching of surrounding structures such as posterior joint capsule, hamstrings, and gastrocnemius [21, 25], so surgeons should be careful not to determine intraoperative mediolateral ligament balance only at extension. Although minimal medial release, i.e., medial stabilizing technique, could result in a trapezoidal gap where lateral gap is larger than medial gap, native knees originally have approximately 3° larger lateral joint laxity compared with medial side both at 0° and 90° flexion [27], and medial joint laxity remains unchanged in severe varus knees, whereas lateral joint laxity increases [15]. In a clinical practice, ligament tension is optimized by adjusting the amount of bone cut, without ligament release. The medial femoral condyle is resected distally to the same thickness as the femoral component and posteriorly 1–2 mm thicker than the femoral component taking into consideration the residual articular cartilage [12]. This procedure usually does not require excessive medial release, and could reproduce near-normal ligament laxity preventing medial and anterior instability after CR-TKA. Moreover, medial stabilizing technique has shown favorable clinical outcomes even with a trapezoidal gap [1, 4, 6, 19, 28–31]. Excessive medial release could lead to kinematically unnecessary anteroposterior femorotibial movement [31–35] as well as patient awareness of instability [24, 36], especially in CR-TKA without a prosthetic post-cam mechanism or congruence between the femoral component and tibial insert [4], which also results in inferior clinical outcomes. Redundant medial release is unnecessary during TKA, and a residual asymmetrical joint gap can be permitted [9, 11].

The present study has several limitations. First, the results might be changed in OA knees with osteophytes. In vivo or cadaveric knees with OA should be used to address this issue. However, intraoperative sequential medial release is ethically unacceptable, and it is difficult to obtain only specimens with knee OA. Osteophyte resection sufficiently increases the medial laxity without PMC and sMCL release [19], and lateral joint laxity is considered greater in varus OA knees [14]. The medial soft tissue is not shorter with a greater preoperative varus deformity [15], and the results in the current study can be applied to OA knees as well. Second, the sample size was small. Since the specimens were donated through the patients’ kindness during their lives, the available samples are limited. However, due to the large effect size, enough power (1-β) of 0.99 was obtained with post hoc power analysis. Third, Thiel-embalmed specimens were used, and the biomechanical properties of soft tissues can be altered. However, this embalming method, developed by Walter Thiel in 1992 [37], preserves in vivo soft tissue flexibility well, unlike the conventional fix preparation, allowing for measurements such as knee ROM and ligament balancing [38]. In practice, the knee has an unrestricted ROM during the examination. Fourth, only the coronal and sagittal laxities were examined. The release of the sMCL reduces tibial internal rotation [14, 39]. The PMC plays a key role in tibial internal rotation, particularly near extension [14, 39–42], and release of the PMC increases rotational angulation [7]. Fifth, only one CR prosthesis was used. Different trends might be observed for different implant designs. Lastly, to measure anteroposterior stability after medial release, the thickness of the polyethylene insert was not changed with the same tension in the lateral compartment because the lateral gap did not change after the medial release.

## Conclusions

Medial soft tissue release also increased tibial anterior translation and medial joint gap, and medial joint gap and tibial anterior translation were significantly correlated. Surgeons should be careful not to create too large medial joint gap and tibial anterior translation in flexion by excessive medial release up to the sMCL for achieving an equal mediolateral joint gap in extension.

## Data Availability

The manuscript has no associated data.
